# 活性氧在血小板活化与血栓形成中的作用机制研究进展

**DOI:** 10.3760/cma.j.cn121090-20250710-00327

**Published:** 2026-03

**Authors:** 若依 沈, 开林 徐, 建林 乔, 萍 付

**Affiliations:** 1 徐州医科大学第一临床医学院，徐州 221002 The First Clinical Medicine College of Xuzhou Medical University, Xuzhou 221002, China; 2 徐州医科大学血液病研究所，徐州 221002 Blood Diseases Institute, Xuzhou Medical University, Xuzhou 221002, China; 3 徐州医科大学附属医院血液科，徐州 221002 Department of Hematology, the Afﬁliated Hospital of Xuzhou Medical University, Xuzhou 221002, China

## Abstract

血小板在动脉粥样硬化及血栓形成中发挥重要作用，其活化受活性氧（ROS）调控。ROS主要来源于血小板内的还原型烟酰胺腺嘌呤二核苷酸磷酸氧化酶（NOX），当糖蛋白Ⅵ（GPⅥ）被激活后，可通过脾酪氨酸激酶（Syk）依赖及非依赖途径诱导ROS生成。此外，我们团队最新研究揭示，蛋白酶激活受体（PAR）通路不仅能激活NOX，还可独立于GPⅥ，诱导线粒体依赖性ROS生成。这一发现拓展了PAR的氧化还原调控路径，提示线粒体或可作为抗血栓新靶点。内源性ROS通过修饰信号蛋白和调控离子通道，参与血小板活化的级联反应及血栓稳定。因此，靶向GPⅥ/PAR相关的ROS生成途径，有望为血栓性疾病提供精准治疗策略。本文综述相关机制，为动脉粥样硬化等疾病的防治提供依据。

## Abstract (en)

Platelets play a crucial role in atherosclerosis and thrombus formation, and their activation is regulated by reactive oxygen species（ROS）. ROS in platelets primarily originates from nicotinamide adenine dinucleotide phosphate oxidase（NOX）. Upon activation of glycoprotein Ⅵ（GPⅥ）, ROS generation can be induced through both spleen tyrosine kinase（Syk）-dependent and Syk-independent pathways. In addition, the protease-activated receptor（PAR）signaling pathway not only activates NOX, but our latest research has revealed that it can also induce mitochondria-dependent ROS generation independently of GPⅥ. This finding expands the redox signaling pathways regulated by PAR and suggests mitochondria as a potential novel target for antithrombotic interventions. Endogenous ROS participate in the cascade of platelet activation and thrombus stabilization by modifying key signaling proteins and regulating ion channel function. Therefore, targeting ROS generation pathways associated with GPⅥ/PAR holds promise for developing precise therapeutic strategies for thrombotic diseases. This review summarizes the underlying mechanisms and provides a theoretical basis for the prevention and treatment of conditions such as atherosclerosis.

血小板在止血和病理性血栓性疾病中起着重要的调控作用。活性氧（reactive oxygen species, ROS）是包括血小板在内的生物细胞在正常氧化活动过程中产生的副产物，主要包含超氧阴离子、羟基自由基、过氧化氢（H_2_O_2_）和次氯酸[1]。血小板的表面受体与配体结合后，可迅速产生ROS。作为重要的信号分子，ROS不仅参与调控血小板活化、功能表达及血栓形成[2]，还与衰老、动脉粥样硬化、心血管疾病、神经退行性疾病、糖尿病、高血压等多种病理生理过程密切相关[3]–[5]。各种来源的抗氧化剂可消除ROS从而减轻血小板活化并抑制血栓形成。本综述总结了调节血小板中ROS产生的途径及其对血小板功能的影响，重点关注血小板表面受体糖蛋白Ⅵ（glycoprotein Ⅵ, GPⅥ）和凝血酶受体依赖性血小板反应，这可为血小板受体激活后ROS的生成机制提供新见解，同时也表明ROS可能是预防血栓形成的潜在靶点。

一、血小板活化与血栓形成

在生理状态下，内皮细胞持续合成并释放前列环素和一氧化氮（nitric oxide, NO），分别通过环磷酸腺苷（cyclic adenosine monophosphate, cAMP）和环磷酸鸟苷（cyclic guanosine monophosphate, cGMP）信号通路阻止血小板活化，使血小板处于静息状态，从而保障血管内环境的稳定与完整[6]。当血管因生理应激或病理因素发生损伤时，内皮下基质中的血管性血友病因子（von Willebrand factor, VWF）与胶原暴露，触发血小板黏附级联反应。其中，血小板膜表面的糖蛋白Ⅰbα（GPⅠbα）特异性识别VWF，而GPⅥ则通过结合胶原，介导血小板的初始锚定与活化。​当血小板表面受体与配体结合后，迅速激活血小板内磷脂酶C（phospholipase C, PLC）、类固醇受体共激活因子（steroid receptor coactivator, Src）等信号通路，通过磷脂酰肌醇-3激酶（PI3K）和蛋白激酶C（protein kinase C, PKC）等下游分子，最终促使整合素αⅡbβ3发生构象改变并获得高亲和力。活化的αⅡbβ3通过其配体结合位点结合纤维蛋白原或VWF，进而调控血小板聚集过程，形成早期血栓[7]。同时，活化的血小板释放二磷酸腺苷（adenosine diphosphate, ADP）、血栓素A2（thromboxane A2, TXA2）等生物活性介质，其与血小板表面P2Y受体及TXA2受体结合，通过G蛋白偶联受体（G-protein coupled receptors, GPCR）信号转导，触发胞内Ca²⁺浓度升高与蛋白磷酸化级联反应，形成正向放大环路，显著增强血小板活化程度[8]–[9]。​在血小板活化的过程中，部分血小板经历膜磷脂翻转，导致带负电荷的磷脂酰丝氨酸（L-A-Phosphatidyl-L-serine, PS）从质膜内叶外翻至表面，为凝血因子Ⅸa-Ⅷa（Tenase复合物）和Ⅹa-Ⅴa（凝血酶原酶复合物）提供组装平台，加速凝血酶生成[10]。生成的凝血酶作为关键效应分子，通过裂解蛋白酶激活受体（protease-activated receptor, PAR）家族成员PAR1和PAR4的N端结构域，暴露出其配体，进而引发GPCR依赖的二次激活[11]。这一正反馈机制不仅强化血小板的聚集能力，更促使血小板释放更多促凝物质，最终驱动血栓形成与稳定。

二、ROS的产生途径

在血小板活化过程中，ROS的产生源自多种酶系统与亚细胞结构，主要包括：还原型烟酰胺腺嘌呤二核苷酸磷酸（NADPH）氧化酶（NOX）、环氧化酶1（cyclooxygenase 1，COX1）、黄嘌呤氧化酶（xanthine oxidase，XO）、线粒体以及内质网细胞色素P450（cytochrome P450，CYP450）。

1. NOX：NOX是血小板内ROS产生的主要来源。目前在血管内细胞中已鉴定出4种NOX家族成员，分别为NOX1、NOX2、NOX4和NOX5。其中，NOX1和NOX2在人和小鼠的血小板中均有表达，并参与细胞内ROS的生成过程[12]。此外，研究表明NOX5同样在血小板中表达，抑制其活性可显著减少ROS的产生[13]。本部分我们主要介绍NOX1和NOX2介导ROS生成的分子机制及在血小板活化过程中的作用方式。

NOX1是血小板响应胶原刺激时诱导ROS生成的关键介质，在信号转导过程中发挥重要作用[14]。当胶原分子与血小板表面的GPⅥ受体特异性结合后，GPⅥ迅速激活，触发Src家族激酶（包括Src和Syk）介导的胞内酪氨酸残基磷酸化级联反应[15]。该磷酸化过程进一步诱导Rac活化，后者作为分子开关，促使NOX1与胞质亚基p22phox、NOXO1、NOXA1和Rac组装形成具有催化活性的NOX1酶复合物。​NOX1酶复合物通过催化电子从NADPH向分子氧的电子转移，生成超氧阴离子并代谢为ROS。NOX1介导的ROS具有多重生物学效应：一方面，ROS可正反馈增强GPⅥ与胶原的结合，并促进TXA₂的合成[14]，进一步促进血小板活化；另一方面，ROS还可激活PI3K/Akt等下游信号通路，协同放大血小板黏附、聚集及分泌功能[16]。

NOX2是血小板在凝血酶刺激下产生ROS的核心组分[14]。当凝血酶与血小板表面的PAR4特异性结合后，PAR4发生构象改变并激活GPCR（如Gq和Gs）信号通路，进而触发Src激酶活化及下游Syk等蛋白的酪氨酸磷酸化级联反应[17]–[18]。与NOX1类似，Rac活化是NOX2激活的关键步骤[15]。活化的Src激酶激活Rac，驱动胞质亚基p40phox、p47phox和p67phox易位并与NOX2组装形成催化复合物[19]。该复合物以NADPH为电子供体，将电子转移至分子氧生成超氧阴离子并代谢为ROS。​NOX2来源的ROS在血小板功能调控中发挥着双重作用：一方面，ROS可激活整合素αⅡbβ3，增强其与纤维蛋白原的结合能力，并诱导血小板颗粒释放及P-选择素表达上调，促进血小板黏附与聚集[19]–[20]；另一方面，ROS可激活JAK/STAT信号通路，通过调控基因表达进一步增强血小板的活化状态[21]。

NOX1和NOX2在血小板活化中表现出不同的作用模式。在GPⅥ激动剂诱导的反应中，NOX1起主导作用，NOX2仅对PAR依赖的血小板活化至关重要[22]。值得注意的是，NOX2缺失不影响ROS生成和血小板活化[23]。此外，我们团队利用p47phox基因敲除小鼠证实，p47phox通过与NOX1/NOX2形成复合物，正向调控GPⅥ和PAR依赖的血小板功能、血栓形成以及ROS生成[24]。

2. COX1：从膜花生四烯酸（arachidonic acid, AA）转化为血栓素A₂（TxA_2_），是驱动血小板活化的关键环节。在磷脂酶 A₂（phospholipase A_2_, PLA_2_）的特异性催化下，膜磷脂中的AA被释放并随即进入COX1代谢通路[15]。血小板含有COX1等酶，这些酶在花生四烯酸代谢过程中会产生过氧化物和其他ROS。COX1催化AA转化为前列腺素G2（prostaglandin G2, PGG2），这一过程会产生过氧化物并释放ROS，如H_2_O_2_[25]。临床研究发现，携带遗传性gp91phox（NOX2的催化核心亚基）缺陷的患者体内，AA介导的血小板超氧阴离子生成通路几乎完全阻断，这一现象进一步证实了该通路在血小板活化调控中的关键作用[15]。

3. XO：XO是一种钼黄素蛋白羟化酶，既可作为氧化酶发挥作用，也可作为还原酶（称为黄嘌呤脱氢酶）发挥作用。XO作为嘌呤代谢途径中的关键酶，催化ATP降解产物次黄嘌呤逐步氧化为黄嘌呤，并进一步将黄嘌呤氧化为尿酸。这一连续氧化过程中伴随超氧阴离子和H_2_O_2_的生成[26]。此外，在缺氧条件下，黄嘌呤氧化酶还参与多种底物的羟基化作用，并能从硝酸盐和亚硝酸盐中生成NO。动物实验表明，XO特异性抑制剂别嘌醇可有效抑制犬类血小板活化，并调节血栓形成过程[27]。临床研究发现，不稳定型心绞痛患者血小板中的XO水平显著高于健康个体，提示心肌缺血状态与血小板ROS生成存在潜在关联[9]。未来可借助XO基因缺陷小鼠模型，进一步验证XO在体内的生物学功能。

4. 线粒体：线粒体作为所有依赖氧化磷酸化（oxidative phosphorylation, OXPHOS）细胞的超氧阴离子的主要来源，在血小板生理与病理过程中扮演双重角色。生理状态下，线粒体产生的ROS是氧化还原信号传导的关键介质，通过耦联线粒体功能与细胞整体生物学过程紧密相连，参与调控缺氧适应、存活机制激活及细胞分化等生理活动[28]；而应激状态下，过量ROS生成会打破氧化还原平衡，引发细胞损伤并推动疾病进展。​

线粒体超氧阴离子主要源于电子传输链（electron transport chain, ETC）的复合体Ⅰ和Ⅲ[29]。生成的超氧阴离子首先被线粒体锰超氧化物歧化酶（manganese superoxide dismutase, MnSOD或SOD2）催化歧化为H_2_O_2_，随后经谷胱甘肽过氧化物酶（glutathione peroxidase, GPx）还原为水。超氧阴离子可通过内膜阴离子通道释放至细胞质，触发“ROS诱导的ROS释放”级联反应，导致邻近线粒体的ROS生成显著增加[9]。​

高度依赖ATP的血小板通过糖酵解和线粒体有氧呼吸维持功能。研究表明，胶原/凝血酶诱导的血小板活化会通过钙离子动员，短暂地提升线粒体膜电位（mitochondrial membrane potential, MMP）与氧化磷酸化水平[29]。其中，MMP的升高与线粒体ROS生成呈正相关，而过度超极化会干扰ETC电子传递，促使电子泄漏并上调超氧阴离子的产生[29]。当MMP丧失时，线粒体通透性转换孔开放，细胞色素c释放至细胞质，参与凋亡依赖的PS暴露过程[15]。​

临床与基础研究证实，线粒体ROS对血小板功能具有多维度调控作用。在镰状细胞病中，线粒体ROS异常升高驱动血小板过度活化，而线粒体解偶联或ROS清除可抑制P-选择素和整合素αⅡbβ3的激活[30]。在糖尿病的研究中，抑制线粒体电子传递链复合体Ⅱ或解偶联氧化磷酸化，可显著降低高血糖诱导的ROS水平，证实线粒体为ROS的主要来源[31]。最新研究显示，线粒体靶向抗氧化剂MitoQ可通过减少ROS生成，抑制血小板的P-选择素和CD63表达，进而抑制胶原、convulxin、PAR1和PMA诱导的血小板聚集、黏附与铺展[32]。这些发现揭示了线粒体ROS在维持血小板基础功能中的重要作用。

5. 内质网细胞色素P450（CYP450）：CYP450酶系统在氧化反应过程中也会生成ROS。在CYP450催化的氧化还原反应中，电子从NADPH通过辅因子（如P450还原酶）转移至CYP450酶，激活氧分子。在此过程中，如果氧化还原反应不完全，部分还原的氧分子可转化为ROS[26]，提示CYP450是ROS的一条产生途径。

三、血小板受体激活介导的ROS生成机制

血小板受体的激活与ROS生成在止血与血栓形成过程中发挥重要作用，不同血小板受体通过独特且互作的机制调控ROS生成，以下从典型受体及线粒体途径阐述ROS的生成机制。

1. GPⅥ介导的ROS生成途径：GPⅥ作为膜受体，与Fc受体γ链形成复合物，当与胶原蛋白等配体结合后，通过诱导免疫受体酪氨酸活化基序（Immunoreceptor tyrosine-based activation motif, ITAM）磷酸化激活，驱动信号复合物组装与血小板活化。静息状态下血小板存在基础水平的ROS，GPⅥ刺激后ROS水平迅速升高[33]。其生成机制中，肿瘤坏死因子受体相关因子4（tumor necrosis factor receptor-associated factor 4, TRAF4）作为GPⅥ结合伴侣，与NOX复合物亚基p47phox相互作用，并协同其他激酶介导信号传导[19]。GPⅥ与配体结合后，酪氨酸蛋白激酶磷酸化PKC-δ，促进p47phox磷酸化，促使NOX复合物产生ROS[34]。该过程呈现双相性：不依赖Syk的爆发期由TRAF4/p47phox/NOX信号轴驱动，而依赖Syk的持续期则由Syk/PLCγ2/NOX信号轴介导[33]。

2. PAR介导的GPCR依赖性ROS生成途径：血小板在血管损伤部位的初始黏附依赖GPⅥ/GPⅠb-Ⅸ-Ⅴ通路[35]，而血栓募集则需凝血酶或ADP等可溶性介质介导的GPCR信号[33]。GPCR与异源三聚体G蛋白（如Gα、Gβ、Gγ）耦联，配体结合后诱导亚基解离并激活效应蛋白。凝血酶与PAR结合后，通过Gq和G12/13等通路引发级联反应[33]。除GPⅥ外，PAR激活也能诱导血小板内ROS生成：凝血酶刺激下，NOX1衍生的ROS生成需要PAR4和GPⅠbα共同参与[36]。

3. 线粒体来源的ROS生成途径：尽管NOX是研究焦点，线粒体亦是ROS的重要来源[37]。研究表明，仅凝血酶刺激可诱导线粒体ROS生成，而CRP刺激则无此效应[38]。线粒体靶向抗氧化剂Mito-TEMPO可消除凝血酶诱导的ROS，而对CRP诱导的ROS无影响[38]；NOX抑制剂apocynin则可抑制凝血酶和CRP诱导的ROS生成[33]。这表明NOX参与GPⅥ和PAR依赖性ROS生成，而线粒体仅参与凝血酶诱导通路，且Ca^2+^是PAR依赖性线粒体ROS生成所必需，敲除相关酶可降低凝血酶刺激下的线粒体ROS水平和血小板活化[33]。

四、ROS在血小板功能调控中的作用及分子机制

在血小板活化的过程中，细胞内ROS作为关键的第二信使，通过多维度调控机制驱动血小板的活化与功能发挥。具体而言，ROS可通过调节血小板受体功能、影响激动剂生物利用度、参与异前列腺素生成及低密度脂蛋白氧化等途径，促进血小板活化[19]。其中，NOX2来源的血小板内ROS能特异性调控凝血酶诱导的P-选择素暴露、整合素αⅡbβ3活化及细胞内钙释放[15]，进一步表明ROS在血小板活化过程中的重要作用。

从作用机制来看，NOX抑制剂与超氧阴离子清除剂可通过修饰整合素αⅡbβ3的巯基，阻断其构象变化并降低纤维蛋白原结合能力，导致血小板聚集功能障碍，这一现象充分表明了ROS在血小板聚集中的重要作用[33]。进一步研究显示，ROS清除剂可有效抑制血小板活化，而ROS诱导剂（如2,3-二甲氧基-1,4-萘醌）则能增强血小板的活化程度[39]；抗氧化剂（如超氧化物歧化酶）还能降低胶原刺激下血小板释放的ADP水平，进一步表明了ROS在血小板活化过程中的关键作用[39]。尽管ROS广泛参与血小板活化，但在不同激动剂诱导的活化过程中，其作用存在明显差异：细胞外抗氧化剂仅能抑制凝血酶诱导的P-选择素表达和αⅡbβ3活化，对GPⅥ激动剂convulxin诱导的反应无抑制作用[19]。此外，ROS还可通过与血小板聚集的天然抑制剂NO发生反应，降低NO的生物利用度，间接增强血小板的活化[13]。

血小板膜糖蛋白（glycoproteins, GPs）的动态调节是止血过程中血小板发挥黏附与促凝活性的关键[40]。其中，金属蛋白酶介导的GPⅥ和GPⅠbα的蛋白水解可降低受体密度，是抑制过度活化的重要机制[41]–[42]。解整合素金属蛋白酶17（ADAM17）主要参与GPⅠbα的脱落[43]，而ADAM10负责GPⅥ的脱落[44]。具体的调控机制是ROS通过氧化半胱氨酸残基激活ADAM家族蛋白酶，促进GPⅥ和GPⅠbα脱落，下调受体在血小板表面的表达水平与活性[45]。进一步支持这一观点的是：ROS清除剂能够抑制血小板受体脱落，在储存过程中，ROS水平较高的血小板呈现出更高的受体脱落率[46]。此外，氧化应激通过p38信号通路激活ADAM17，诱导GPⅠbα和GPⅤ脱落，致使血小板功能活性降低[47]。我们团队的研究亦发现，免疫性血小板减少症（ITP）患者的血浆可诱导血小板内ROS生成，伴随ADAM17表达上调及GPⅠbα脱落，而ROS清除剂（N-乙酰-L-半胱氨酸）可抑制该过程，且此类ROS主要来源于NOX和线粒体[48]。

五、血小板ROS靶向治疗策略的研究进展

鉴于ROS在血小板活化与血栓形成进程中扮演的核心调控作用，研发靶向ROS生成的治疗策略已成为抑制血小板功能、防控血栓性疾病的重要方向。大量研究证实，抗氧化剂可通过抑制血小板活化与聚集，在血栓性及心血管疾病治疗中展现出潜力。然而，由于ROS在细胞信号转导及生理过程中具有不可或缺的作用，全身性靶向ROS的治疗策略需谨慎应用，以避免对其他细胞产生脱靶效应并引发不良反应。因此，开发高选择性的靶向方案（如通过阻断NOX复合物，精准抑制血小板受体结合诱导的ROS生成）已成为当前研究的迫切需求。

1. 靶向血小板表面受体的精准干预策略：血小板表面受体与配体的结合触发ROS级联反应，因此靶向该类受体成为抑制ROS产生的关键途径。本团队前期研究表明，核苷酸结合寡聚化结构域2（nucleotide-binding oligomerization domain 2, NOD2）拮抗剂GSK699可显著抑制胶原或CRP诱导的血小板活化及ROS生成[49]。体内实验进一步证实，GSK699在有效抑制动脉血栓形成的同时，未增加出血风险，凸显其作为抗血小板药物的安全性优势[49]。在凝血酶受体研究中发现，使用SCH79797或BMS200261阻断PAR1无法抑制凝血酶诱导的ROS产生；而PAR4拮抗剂tcY-NH2预处理则可完全消除该过程产生的ROS，这一发现揭示了PAR4在凝血酶诱导ROS生成中的决定性作用[36]。

2. 细胞内信号通路的靶向调控机制：调控介导ROS生成的细胞内信号通路是抑制ROS产生的重要策略。NOX抑制剂2-乙酰吩噻嗪可选择性抑制胶原或CRP（但对凝血酶无效）诱导的αⅡbβ3、PKC和Syk的激活，以及超氧阴离子的生成。在动脉血流环境中，氧化低密度脂蛋白（ox-LDL）通过CD36受体介导的血小板过度活化依赖于ROS生成，并在CD36缺陷型血小板中显著减弱。深入研究表明，ox-LDL/CD36诱导的ROS生成依赖于Src/PKC/NOX2信号轴，为血脂异常相关疾病中的ROS介导的血栓事件提供了靶向CD36或PKC信号通路的治疗新方向[19]。此外，Src家族激酶和整合素αⅡbβ3的抑制虽然不影响GPⅥ依赖的ROS生成，但PI3K或Syk的抑制可部分减少该过程的ROS生成[50]。进一步观察研究发现，GPⅥ依赖的ROS生成存在双相模式：初始2 min内为不依赖Syk的爆发阶段，随后进入依赖Syk的持续生成阶段[50]。

3. 缺氧诱导因子（hypoxia-inducible factors，HIF）的调控作用：HIF在缺氧环境下的细胞功能调节中起关键作用。研究表明，HIF-1α在K562细胞中可负向调控ROS生成[51]。我们团队利用HIF脯氨酰羟化酶抑制剂IOX-2的研究表明，IOX-2可上调血小板中HIF-1α的表达，进而降低NOX1表达与ROS生成量，同时伴随p38、细胞外信号调节激酶1/2（ERK1/2）、Akt和PKCδ磷酸化水平的下降[52]。此外，IOX-2会损害机体止血功能与血栓形成过程，这表明HIF-1α有望成为调节ROS生成、治疗血栓性疾病的新靶点[53]。

4. 现有药物的ROS靶向治疗潜力：部分临床药物展现出靶向ROS生成的抗氧化特性。以阿托伐他汀为例，其不仅具有调脂作用，还具备抗血小板和抗氧化活性。在高胆固醇血症患者中，阿托伐他汀治疗可显著降低尿异前列腺素和血清NOX2水平，抑制血小板活化，并减弱NOX2激活以及血小板p47phox、Rac-1和PKC的磷酸化，提示其可通过干扰氧化还原信号抑制血小板功能，减少NOX衍生的ROS生成。体外实验进一步证实，阿托伐他汀以剂量依赖性抑制血小板活化、钙动员、NOX2活性及PKC和Rac1的磷酸化。此外，我们的研究还发现，富马酸二甲酯[54]、苦参碱[34]、秋水仙碱[55]可通过抑制血小板功能、降低ROS生成或阻断GPⅥ刺激诱导的血小板活化与促凝活性，为抗血栓治疗药物拓展提供了理论依据。

六、总结与展望

血小板的氧化还原状态与其生理功能形成精密调控网络，在血栓形成过程中发挥关键作用。血管损伤初期，血小板GPⅥ与胶原等配体特异性结合，迅速触发ROS的爆发式生成。这一过程通过双重机制驱动血栓形成：一方面，ROS通过氧化修饰胞内信号蛋白（如PLCγ2-PKC通路），激活血小板从静息状态转为活化状态；另一方面，凝血酶激活GPCR介导的信号转导以ROS作为第二信使，推动血小板活化的级联放大及血栓结构稳定化。二者协同调控下，ROS通过氧化巯基修饰、调节离子通道活性等机制，精确调控血小板α颗粒分泌、整合素αⅡbβ3活化及血小板-血小板/血小板-白细胞黏附互作。

针对ROS在血栓形成中的主导地位，开发选择性靶向策略成为治疗血栓性疾病的前沿方向。与全身性抗氧化治疗相比，靶向血小板特异性受体或衔接蛋白可规避脱靶效应。以TRAF4为例，其通过特异性激活NOX复合物调控GPⅥ依赖性ROS生成。靶向TRAF4-NOX信号轴既能有效抑制血小板过度活化，又可最大限度保留ROS在止血过程中的生理性功能。此外，针对PAR、整合素αⅡbβ3等关键靶点的研究证实，通过干扰受体-配体相互作用或阻断下游信号转导，可实现对ROS生成的精准调控，为血栓性疾病的精准治疗提供新思路。

当前研究仍面临诸多挑战。ROS生成的调控网络呈现高度复杂性：同一刺激可激活多条ROS生成途径，不同亚型NOX在血小板中的表达差异显著，且ROS与其他信号分子（如NO、Ca²⁺）存在精确的交互调节。特别是GPⅥ与PAR介导的ROS生成在不同病理环境下的功能异质性，以及NOX复合物激活的分子机制细节，仍需深入探究。未来研究需结合单细胞测序、活细胞成像等前沿技术，系统解析血小板氧化还原信号的动态调控图谱，为开发兼具安全性与有效性的抗血栓药物提供理论支撑。
